# Dual-functional ultraviolet photodetector with graphene electrodes on AlGaN/GaN heterostructure

**DOI:** 10.1038/s41598-020-79135-y

**Published:** 2020-12-16

**Authors:** Bhishma Pandit, E. Fred Schubert, Jaehee Cho

**Affiliations:** 1 School of Semiconductor and Chemical Engineering, Jeonbuk National University, Jeonju, 54896 Republic of Korea; 2 Department of Electrical, Computer, and Systems Engineering, Rensselaer Polytechnic Institute, Troy, NY 12180 USA

**Keywords:** Materials science, Optics and photonics

## Abstract

A dual-functional ultraviolet (UV) photodetector with a large UV-to-visible rejection ratio is presented, in which interdigitated finger-type two-dimensional graphene electrodes are introduced to an AlGaN/GaN heterostructure. Two photocurrent generation mechanisms of photovoltaic and photoconductive dominances coexist in the device. The dominance of the mechanisms changes with the induced bias voltage. Below a threshold voltage, the device showed fairly low responsivities but fast response times, as well as a constant photocurrent against the induced bias. However, the opposite characteristics appeared with high bias voltage. Specifically, above the threshold voltage, the device showed high responsivities with additional gain, but slow rise and recovery times. For instance, the responsivity of 10.9 A/W was observed with the gain of 760 at the induced bias voltage of 5 V. This unique multifunctionality enabled by the combination of an AlGaN/GaN heterostructure with graphene electrodes facilitates the development of a single device that can achieve multiple purposes of photodetection.

## Introduction

Semiconductor-based ultraviolet (UV) photodetectors with very high sensitivity, stability, speed, and signal-to-noise (S/N) ratio have attracted much attention in recent years for their potential applications in environmental monitoring and security systems. Such applications include flame monitoring, missile plume detection, ozone layer monitoring, biological and chemical analysis, and secure optical communication in outer space^1–4^. As demand and expectation have begun to exceed the performance of conventional UV photodetectors, the development of novel UV photodetectors with special functions or multiple functions has become necessary^5,6^. Smart, flexible, and self-powering characteristics, as well as intelligence, are expected to be essential for next-generation UV photodetectors in the internet-of-things (IoT) era^7,8^.

In order to develop high-performance UV photodetectors, various material- and structure-based approaches have been investigated and published^2,4,9,10^. First, regarding materials, GaN and its alloys with Al and In are highly promising for both visible-blind and solar-blind UV detection without requiring bulky optical filters or cooling systems, unlike Si-based UV photodetectors^11^. Special attention has been paid to AlGaN ternary semiconductors, in which, by tuning the Ga and Al compositions, the bandgap energy can be varied from 3.4 eV (GaN) to 6.2 eV (AlN), thereby covering the UVA to UVC spectral region. In addition to the use of a single layer of nitride semiconductors for photodetector applications, an AlGaN/GaN heterostructure can provide additional benefits of high-speed and high-gain operation. Such a heterostructure intrinsically forms a two-dimensional electron gas (2DEG) at the interface between AlGaN and GaN; this simultaneously provides both high carrier density and mobility^12–14^. In fact, AlGaN/GaN heterostructures are essential platforms for high-electron-mobility transistor technology for next-generation high-power and high-frequency devices^15,16^.

Graphene, a two-dimensional (2D) hexagonal lattice array of carbon atoms, is attractive for applications as transparent conductive electrodes because of its high electrical and thermal conductivities, transparency, and mechanical strength^17–20^. Previous studies have confirmed that the graphene/GaN contact is stable under high-temperature and high-radiation operations, indicating that graphene electrodes are appropriate for applications in fire alarms and space exploration devices, which operate in extremely harsh conditions^21^. Our previous studies demonstrated the successful implementation of graphene and reduced graphene oxide as transparent electrodes on AlGaN layers with various Al mole fractions, showing sharp cut-off wavelengths at the energy bandgaps of AlGaN^22,23^. In this regard, the combination of graphene electrodes and AlGaN/GaN heterostructures may yield an excellent UV photodetector, considering the high transparency of graphene in the UV spectral region and the high electrical conductivities of graphene and 2DEG, which would benefit efficient charge collection in the photodetector.

Secondly, in structural approaches, various photodetector structures have been employed, including a metal–semiconductor–metal (MSM) photodiode, p–n and p–i–n photodiodes, an avalanche photodiode, a photoconductor, and a phototransistor^4,24^. Among them, the MSM structure with back-to-back Schottky contacts provides a low dark current, high S/N level, fast response speed, and compatibility for integration with other circuit components, particularly via simple fabrication methods^25,26^. The MSM-type structure can be fabricated in one step of electrode deposition on a semiconductor material without any deteriorations of device performance from the complicated processes of etching and diffusion, as well as those from the asymmetric traveling lengths of electrons and holes before reaching an electrode.

**Figure 1:**
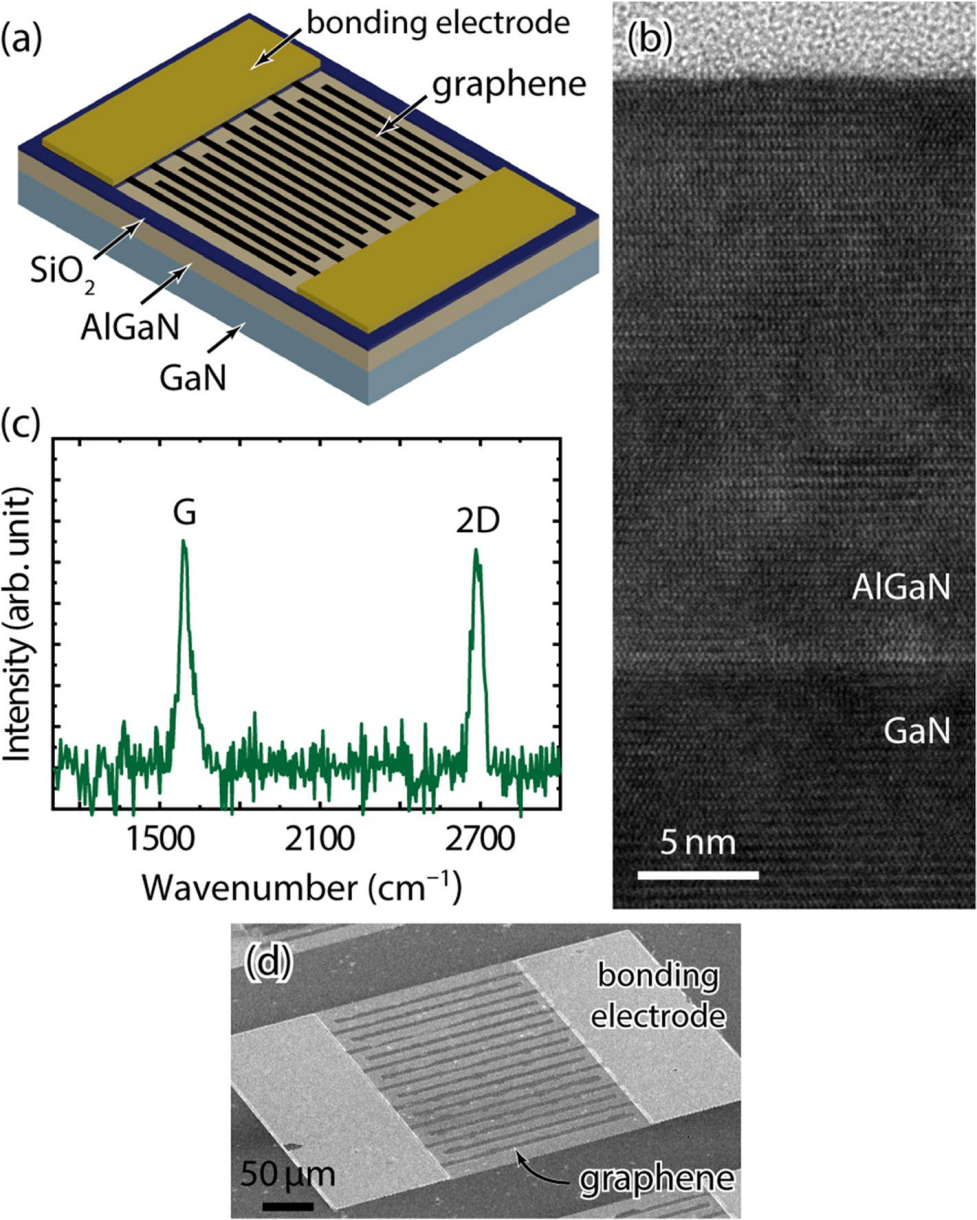
Structural analysis. (**a**) Schematic of the Gr/AlGaN/GaN photodetector structure. (**b**) Transmission electron microscope image of AlGaN/GaN heterostructure. (**c**) Raman spectrum of Gr/AlGaN/GaN. (**d**) Scanning electron microscope image of the fabricated device.

In this study, we present a dual-functional (i.e., either high-speed or high-gain) UV photodetector comprising an AlGaN/GaN heterostructure and a graphene electrode (hereafter, Gr/AlGaN/GaN). The photodetectors presented here have an MSM-type structure, as shown schematically in Fig. 1a, and were investigated under broad wavelength range illumination and various bias voltages. We found that two photocurrent generation mechanisms of photovoltaic and photoconductive work together in the devices; these dual functionalities can be easily switched by varying the induced bias voltage on the device.

## Results

The transmission electron microscopy (TEM) image in Fig. 1b shows a strain-unrelaxed epitaxially grown AlGaN layer on the thick GaN underlayer. The growth of the AlGaN/GaN heterostructure spontaneously initiated the formation of a 2DEG with the mobility of 1980 cm^2^/V·s and the sheet carrier density of 5.6 × 10^12^ cm^−2^ at room temperature. Figure 1c shows the Raman spectroscopic profile of the Gr/AlGaN/GaN. The well-known G and 2D peaks of graphene are found at the wavenumbers of about 1590 and 2700 cm^−1^, respectively^27^. The peak intensity ratio of I_2D_/I_G_ is less than 1, indicating the presence of multilayered graphene^28^. The final MSM-like device structure of Gr/AlGaN/GaN stacks has eight pairs of interdigitated finger electrodes of 10 µm in width, 10 µm in spacing, and 300 µm in length, as shown in the scanning electron microscope image of Fig. 1d.

**Figure 2:**
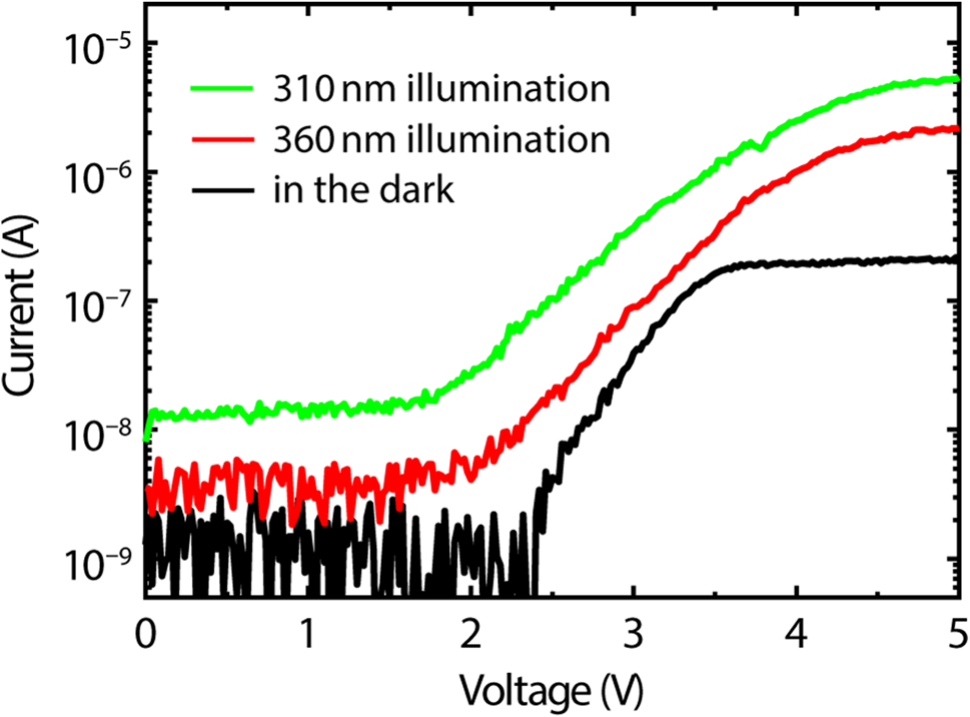
Current–voltage characteristics of the Gr/AlGaN/GaN photodetector under various illumination conditions.

Figure 2 shows the current–voltage (I–V) characteristics of a Gr/AlGaN/GaN photodetector in the dark and under illumination at 360 and 310 nm. A close examination of the I–V curve in the dark (black line in Fig. 2) shows typical diode behavior, i.e., an extremely low dark current of ~ 1 nA below a threshold voltage, a gradual increase in current above the threshold voltage, and then current saturation due to the electron saturated velocity. Because of the Schottky-like contact property between graphene and AlGaN (see “Supplementary Information” for more details on the Schottky barrier height), a threshold voltage of about 2.4 V exists before current increases exponentially as a function of bias. As shown in Fig. 2, photocurrents are generated under illumination at both 360 and 310 nm, with distinct photoresponsivity over the entire bias voltage range. The shorter incident wavelength yields a higher photocurrent with faster turn-on (i.e., a lower threshold voltage). Absorption of the high-energy photons may lower the energy barrier of the Schottky-like contact, thus lowering the threshold voltage. Overall, the shapes and tendencies of the three I–V curves are similar, except current saturation does not occur for the photocurrents under 360- and 310-nm illumination.

**Figure 3:**
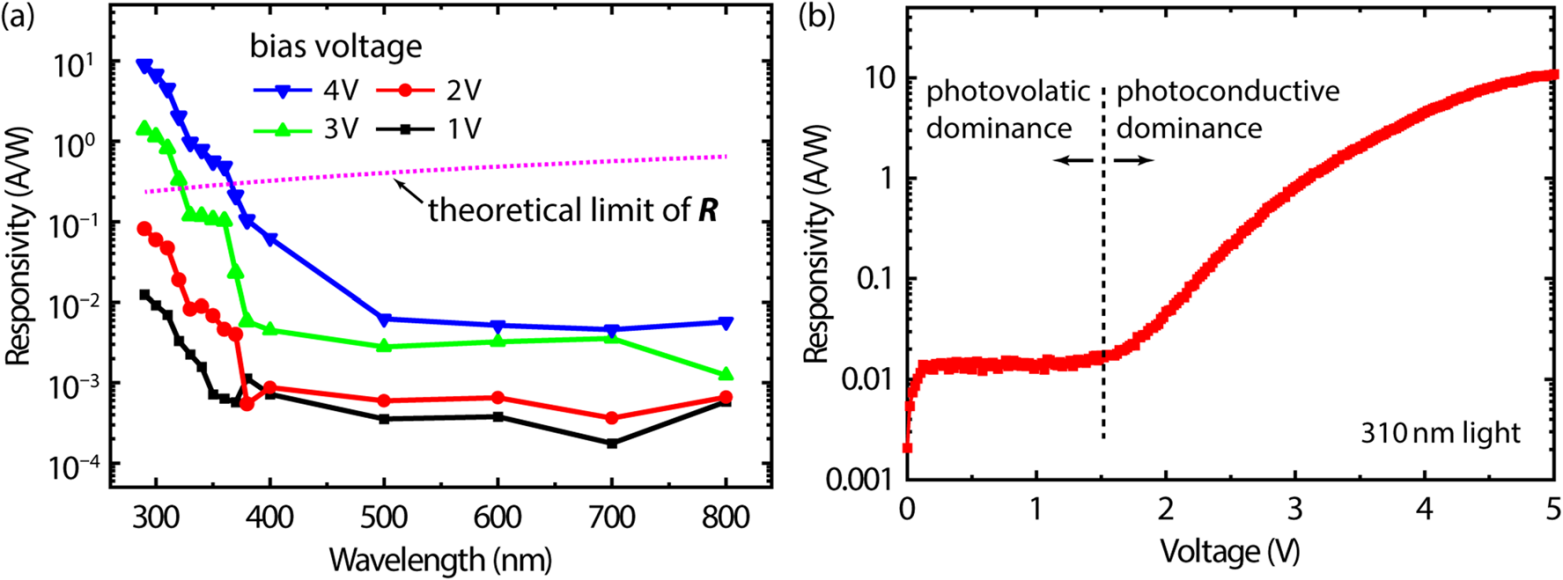
Photoresponse. (**a**) Spectral photoresponse of the Gr/AlGaN/GaN photodetector under various bias voltages. (**b**) Responsivity as a function of induced voltage under 310-nm illumination. Note the logarithmic scale of the responsivity axis.

As a representative figure-of-merit of a photodetector, the responsivity (R) was calculated by using the relation^29^:

1 $$R=({I}_{P}-{I}_{D})/\Phi$$

where I_D_ is the dark current, I_P_ is the photocurrent, and Φ is the incident optical power, all of which are easily measurable values. Measurements of responsivity as a function of wavelength are performed at various bias conditions, as shown in Fig. 3a. Each point is the average value of measurements from multiple devices. These data demonstrate a high UV-to-visible rejection ratio, showing low responsivity in the visible region but increasing responsivity as the incident wavelength enters the UV spectral region, with an exponential increase with further decreases in wavelength. Another important observation in Fig. 3a is the dependency of the responsivity on the induced bias. At low bias voltages (i.e., 1 and 2 V), the responsivities have low absolute values; indeed, they are below the theoretical limits of responsivity for devices in the photovoltaic regime. Details of the two different photocurrent generation mechanisms of a photodetector follow in the next paragraph. In order to examine the theoretical limit of responsivity in detail, the responsivity of Eq. (1) is rewritten as follows^29^:

2 $$R=\eta q/h\upsilon \approx \eta \lambda /1.24$$

where η is the quantum efficiency, q is the elementary charge, h is Planck’s constant, and υ and λ are respectively the frequency in Hz and wavelength in μm. According to this equation, R has a theoretical limit (i.e., R at η = 100%) at each wavelength, which can be reached when the device involves no gain mechanism. The dotted line in Fig. 3a shows the limit as a function of the wavelength; the responsivities of the device when operated at 1 and 2 V are always below this line. However, the responsivities at the bias voltages of 3 and 4 V are higher than those at 1 and 2 V, and they exceed the theoretical limit at the short-wavelength region. For example, under 310-nm illumination, the device has significantly higher responsivities of 0.8 and 4.5  A/W at the bias voltages of 3 and 4 V, respectively, in contrast to those of 0.01 and 0.05 A/W at the bias voltages of 1 and 2 V, respectively. The higher responsivities exceed that indicated by the 100% quantum efficiency limit of 0.25 A/W at 310 nm. Therefore, these high responsivities indicate the presence of a gain mechanism in the photodetector device.

Now let us discuss two different photocurrent generation mechanisms that can exist in semiconductor photodetectors^29^. First, a junction of two dissimilar materials can yield the photovoltaic effect, in which the dissimilarity can be different dopant types (homojunction), different materials themselves (heterojunction), and the contact between metal and semiconductor (Schottky-type)^4,30^. As photons impinging on the junction are absorbed, the absorbed photons produce free charge carriers within the depletion region. The existing electric field then causes the charge carriers to move apart, resulting in current flow in an external circuit. The most common photovoltaic-effect-based photodetectors are photodiodes and phototransistors. Second, photoconductivity (i.e., the photoconductive effect) occurs when photons impinge on semiconducting materials, causing the excitation of electrons from the valence band to the conduction band, and thus affecting the material conductivity^31,32^. This photoconductive effect is widely utilized in electromagnetic radiation sensors, photoconductors, light-dependent resistors, and photoresistors. CdS and CdSe are typical semiconductors used in photoconductive-effect-based photodetectors and sensors^33,34^. Generally speaking, photodetectors in these two categories have certain strengths and weaknesses. For example, in comparing a photodiode and a photoconductor, a photodiode operated under the photovoltaic effect has device characteristics as follows^35,36^: (1) relatively low responsivity limited by the wavelength [refer to Eq. (2)], (2) high detectivity, (3) fast response time, and (4) ideally no dependence of responsivity on external bias. However, a photoconductor operated under the photoconductive effect has the opposite device characteristics, as follows: (1) relatively high responsivity due to an internal gain process, (2) low detectivity, (3) slow response time, and (4) strong dependence of responsivity on external bias. If only the respective strengths of these two device types could be combined, the resulting device would be great for diverse and versatile photodetectors.

Figure 3b shows the responsivity of a typical Gr/AlGaN/GaN photodetector as a function of the bias voltage under 310-nm illumination. At low bias voltage (< 1.5 V), the responsivity is nearly constant (e.g., 0.014 A/W at 1 V) with a weak dependency on bias voltage, which is an obvious indication that the device is operating in a manner dominated by the photovoltaic effect. Above 1.5 V, the responsivity begins to increase, reaching 10.9 A/W at the bias voltage of 5 V, thus showing a strong dependency on the bias voltage. Note that signs of the two different photocurrent generation mechanisms aforementioned exist in a single device, revealing their specific device characteristics based upon the induced bias conditions. This dual functionality is enabled by the combination of the AlGaN/GaN heterostructure and 2D graphene electrode. Graphene typically forms a Schottky contact on AlGaN, but the Schottky barrier height, or the energy barrier between graphene and AlGaN, is not stiff because of the inhomogeneity of Schottky barriers and the surface-state pinning; therefore, the barrier is easily overcome by the induced bias voltage. Meanwhile, the 2DEG of the AlGaN/GaN heterostructure provides a superior platform to multiply charge carriers excited by light absorption. In other words, the photovoltaic regime is maintained as long as the Schottky-like contact of graphene/AlGaN is valid. After the energy barrier of this contact is punched through at the threshold voltage, the current path from one graphene electrode to the AlGaN/GaN heterojunction via 2DEG and then to the other graphene electrode behaves like a conductor. Therefore, a photocurrent gain (G) occurs and works to increase the primary photocurrent, implying the shift from the photovoltaic to photoconductive regime. Equation (1) can be rearranged to express I_P_ with G as follows^29^:

3 $${I}_{P}-{I}_{D}=R\Phi G\approx R\Phi \tau /{t}_{n}$$

where τ is the minor-carrier lifetime and t_n_ is the major-carrier transit time. Here, t_n_ is inversely proportional to the induced bias voltage as ${t}_{n}=l/({\mu }_{e}E),$ where *l* is the photoconductor length, *E* is the electric field, and μ_e_ is the electron drift mobility, showing a high dependency on bias. For instance, a simple calculation comparing the photocurrents at 1 and 5 V shows that the gain of the device increases to 790 under 310-nm illumination as the induced bias voltage is changed from 1 to 5 V. The value is comparable to that reported previously by Satterthwaite et al. with an MSM-type photodetector comprising an AlGaN/GaN heterostructure and a Pd/Au electrode^37^.

**Figure 4:**
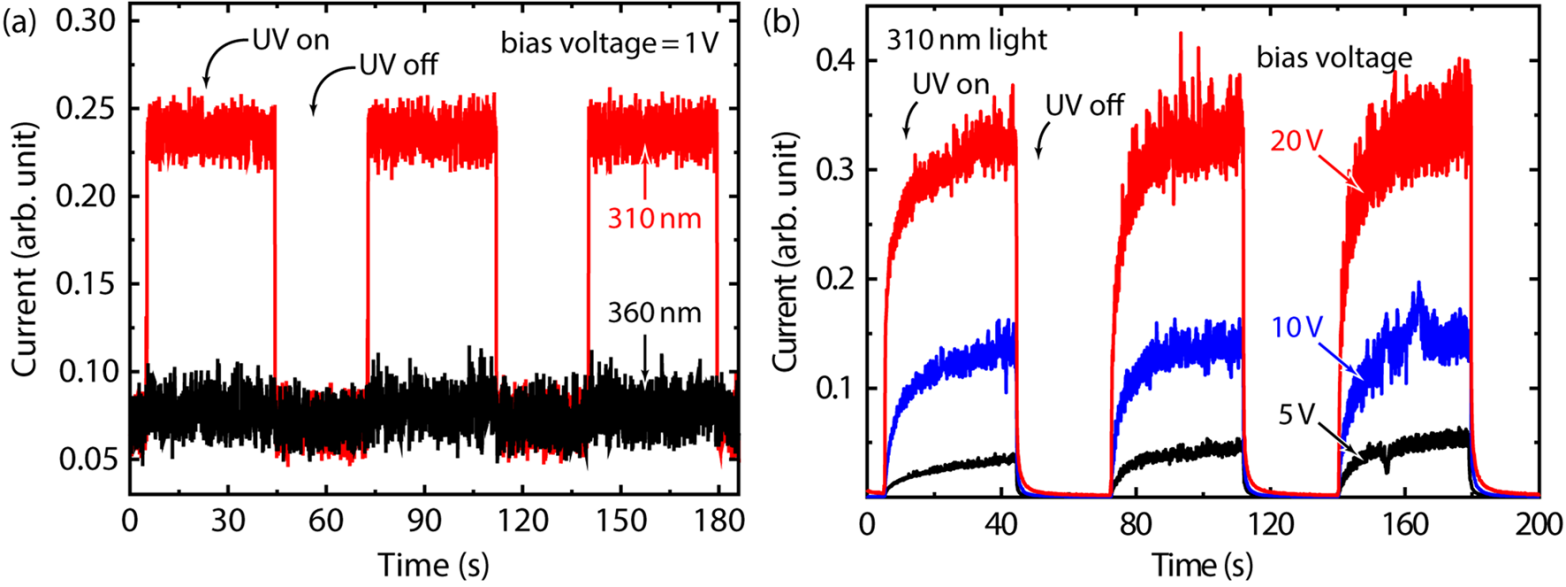
Temporal photoresponse of the Gr/AlGaN/GaN photodetector (**a**) at the bias voltage of 1 V under 310- and 360-nm illumination, (**b**) at the bias voltages of 5, 10, and 20 V under 310-nm illumination.

The response speed of a photodiode is mainly limited by the carrier transit time in the depletion region, if the external effects such as capacitances and external resistors are excluded from consideration. On the other hand, the response speed of a photoconductive detector is influenced not only by the carrier transit time, but also by the minor-carrier lifetime. The longer carriers live, the higher they contribute to photocurrent; however, a long carrier lifetime consequently leads to a slow response speed. Therefore, for strong evidence of the coexistence of the two photocurrent mechanisms in one device, the temporal photoresponses were measured while changing the induced bias voltage. Figure 4a shows the temporal photoresponse of the Gr/AlGaN/GaN photodetector at 1 V with 360- and 310-nm illumination. Note that the bias condition of 1 V is part of the photovoltaic regime, as shown in Fig. 3b. No photoresponse occurs under 360-nm illumination, but the data are shown for comparison purposes. Under 310-nm illumination, a sharp on/off effect in the photocurrent is observed, with response and recovery times of < 0.1 s. For high induced bias voltages, Fig. 4b shows the temporal photoresponses measured under 310-nm illumination. The higher bias voltages from 5 to 20 V show significantly larger photocurrents, confirming the strong dependency on bias voltage. Contrary to the result in Fig. 4a, the photoresponses at the bias voltages of 5, 10, and 20 V show slow gradual rises and recoveries against on/off operation. Again, these slow photodetector responses are characteristic of the photoconductive regime, including the 5-, 10-, and 20-V bias conditions. Temporal measurements at low- and high-bias conditions thus represent well the specific characteristics of the photovoltaic and photoconductive effects, verifying that two different photocurrent generation mechanisms occur in the device, which can be selected by the induced bias voltage.

As another key parameter of photodetectors, the detectivity (D) shows how much minimum signal can be detected by the photodetector. The detectivity can be calculated by using the simplified equation^38,39^:

4 $$D\approx \frac{{A}^\frac{1}{2}R}{{(2q{I}_{D})}^\frac{1}{2}}$$

where A is the junction area and the other symbols have their usual meanings as previously mentioned. The calculation showed the detectivities of 1.02 × 10^10^ and 6.19 × 10^11^ cmHz^1/2^ W^–1^ at the bias voltages of 1 and 4 V, respectively, under 310 nm (see “Supplementary Information”, Table S1). Although the detectivities in the photoconductive regime show higher values than those in the photovoltaic regime, the increase in detectivity is not comparable with the increase in responsivity because the large dark current in the photoconductive regime.

## Discussion

In conclusion, a dual-functional Gr/AlGaN/GaN UV photodetector with a clear UV-to-visible rejection ratio is presented. The photodetector shows two photocurrent generation mechanisms in a single device; the mechanisms can be selected by the induced bias voltage. Under low bias conditions, i.e., below the threshold voltage, the device showed relatively low responsivities, but fast response times and constant photocurrent against the induced bias. At high-bias conditions, i.e., above the threshold voltage, the device showed high responsivities with additional gain, but a slow response time and a strong dependency of the photocurrent on the induced bias. Depending on the bias voltage induced on the photodetector device, the dominant device features of the photodetector can be easily tuned; thus, a single device can be adjusted to meet any purpose of UV photodetection. This unique multifunctionality enabled by the combination of an AlGaN/GaN heterostructure and a 2D graphene electrode is expected to be highly useful to the development of UV photodetectors for the coming IoT era.

## Methods

### Device fabrication

Devices were fabricated on an AlGaN/GaN heterostructure grown on a Si substrate by metalorganic chemical vapor deposition (MOCVD). In detail, after a 5-μm-thick layer of undoped GaN underlayer was grown on a p-type 6-in. Si (111) substrate, a 21-nm-thick AlGaN layer with a 20% Al mole fraction and a 3-nm-thick GaN cap layer were subsequently grown on the GaN underlayer. Multilayered CVD-grown graphene was transferred by a polymethyl methacrylate (PMMA)-assisted wet transfer method^40^. In brief, PMMA was spin-coated on a graphene-coated Cu foil and subjected to soft baking at 180 °C for 5 min. The PMMA/graphene/Cu stack was then treated with ammonium persulfate solution for 7 h to etch away Cu. In sequence, the separated graphene/PMMA stack was transferred to the surface of the AlGaN/GaN heterostructure sample. To improve the adhesion between the graphene and AlGaN semiconductor, the sample was baked at 200 °C for 3 h on a hot plate. Next, PMMA was removed by an acetone treatment at 60 °C. After a 100-nm-thick SiO_2_ layer was deposited by a plasma-enhanced CVD system, an interdigitated finger structure was introduced by using a standard photolithographic technique and wet-etching of buffered oxide etchant. Probing metal electrodes of Ni/Au were deposited by electron-beam evaporation and the graphene layer in unnecessary areas was etched away by O_2_ plasma.

### Measurements

The I–V characteristics of the Gr/AlGaN/GaN photodetectors were measured by a source measurement unit (SMU, Keithley 4200) equipped with a dark-room probe station. The wavelength-dependent and temporal responsivities were measured by combining a Bentham switching monochromator and the SMU. The illumination power of the switching monochromator was calibrated by a Newport optical power meter. All presented measurements were taken at room temperature.

## Supplementary Information

Supplementary information.
