# Supplementary material for: Dual-functional ultraviolet photodetector with graphene electrodes on AlGaN/GaN heterostructure
Source: Sci Rep. 2020 Dec 16;10:22059. doi: 10.1038/s41598-020-79135-y (PMC7745009; doi:10.1038/s41598-020-79135-y)
Supplement: Supplementary file 1 — Supplementary information. [file 41598_2020_79135_MOESM1_ESM.docx]

Supplementary Information

**Dual-functional ultraviolet photodetector with graphene electrodes on AlGaN/GaN heterostructure**

Bhishma Pandit^1^, E. Fred Schubert^2^ & Jaehee Cho^1,^*

^1^School of Semiconductor and Chemical Engineering, Jeonbuk National University, Jeonju 54896, Republic of Korea

^2^Department of Electrical, Computer, and Systems Engineering, Rensselaer Polytechnic Institute, Troy, NY 12180, United States

*Correspondence and requests for materials should be addressed to prof. Jaehee Cho (e-mail: jcho@jbnu.ac.kr)

**Schottky barrier height (SBH) measurement of the Gr/AlGaN/GaN contact**


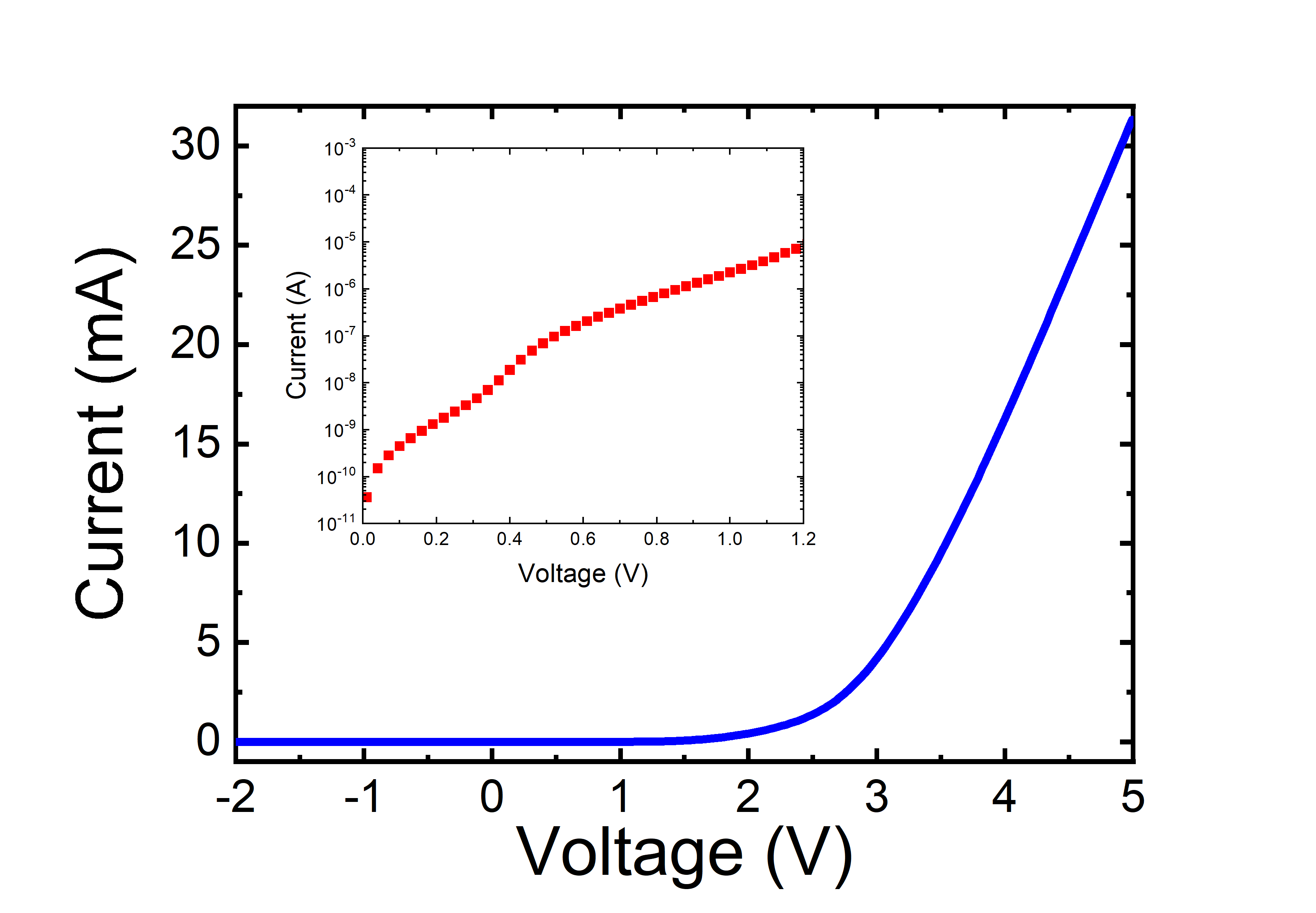


**Fig. S1** I–V characteristics of the Gr/AlGaN/GaN Schottky diode. The inset shows the sub-threshold region in a semi-logarithmic scale.

A Schottky diode, in which Ti/Al/Ni/Au and graphene were used as an ohmic and Schottky contact, respectively, was fabricated on the AlGaN/GaN heterostructure. As shown in the inset of Fig. S1, measurement of the relationship of the logarithmic current vs. the voltage of a Schottky diode and determining the y-axis intercept by extrapolating the linear part of the curve enable the saturation current (*I*_0_) to be obtained. This makes it possible to calculate the Schottky barrier height (SBH, Φ_B_) of a metal–semiconductor junction by employing the well-known thermionic emission model as follows:

$I_{0}=AA^{*}T^{2} exp\left( -\frac{q\Phi_{B}}{k_{B}T} \right)$ (1)

where *q* is the elementary charge, *k*_B_ is the Boltzmann constant, *T* is the absolute temperature, *A* is the Schottky area of the diode, and *A*^*^ is the Richardson constant for a semiconductor. (here, *A* = 2000 μm^2^ and *A*^*^ = 28.77 Acm^–2^K^–2^ for Al_0.2_Ga_0.8_N)

The measurement yielded approximately 0.73 eV for the SBH of the graphene/AlGaN/GaN Schottky diode at room temperature.

**Current–voltage characteristics of the Gr/AlGaN/GaN heterostructure**

**
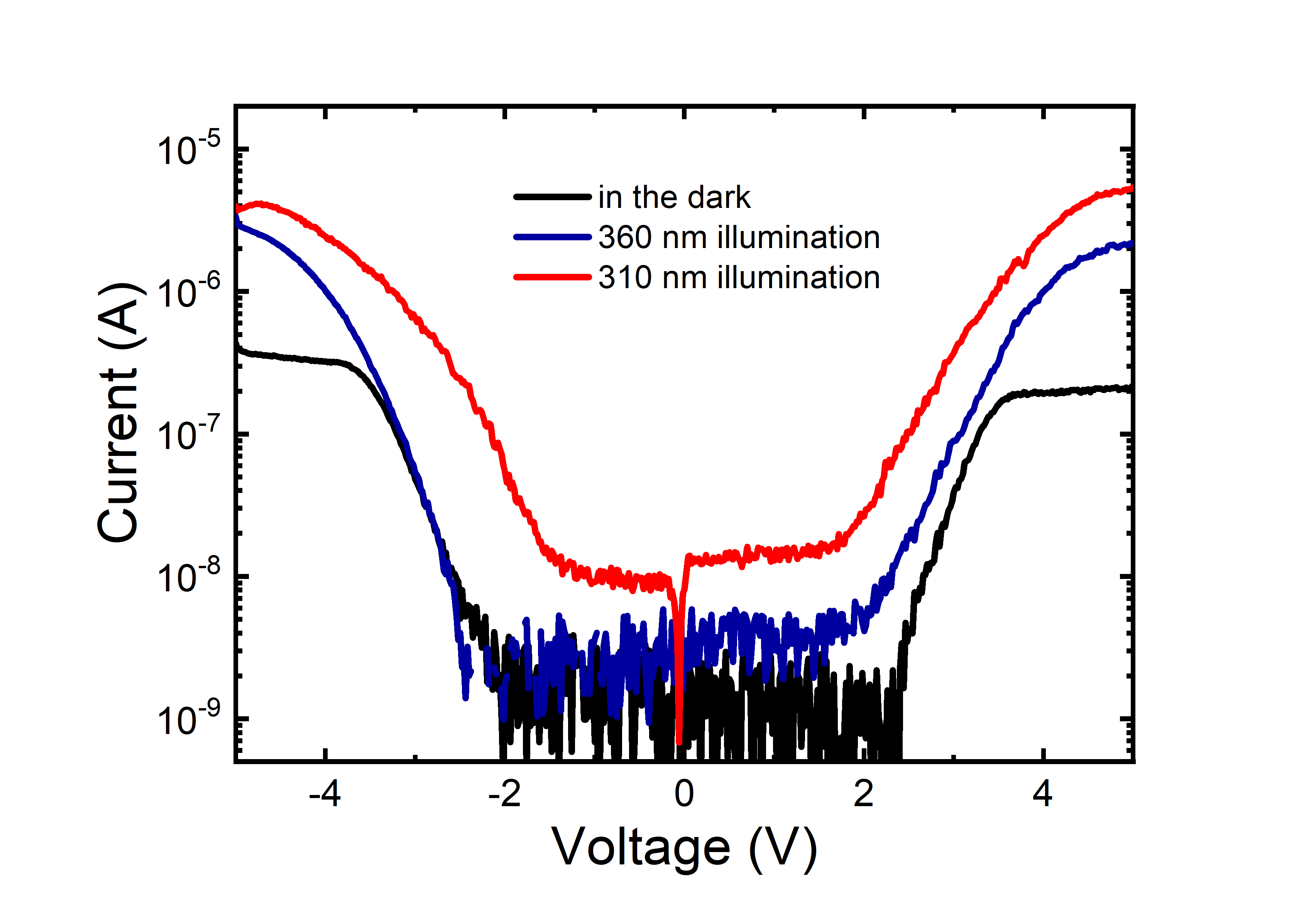
**

**Fig. S2** I–V characteristics of the Gr/AlGaN/GaN photodetector under various illumination conditions.

**Detectivity (*D*) of the Gr/AlGaN/GaN photodetector**

**Table S1** Detectivity (*D*) of the Gr/AlGaN/GaN photodetector at various illumination conditions and bias voltages.

| Bias voltage | ***D*** under 360 nm (cmHz^1/2^W^–1^) | ***D*** under 310 nm (cmHz^1/2^W^–1^) |
| --- | --- | --- |
| 1 V | 9.30 × 10^8^ | 1.02 × 10^10^ |
| 2 V | 5.87 × 10^9^ | 5.96 × 10^10^ |
| 3 V | 3.32 × 10^10^ | 2.65 × 10^11^ |
| 4 V | 6.63 × 10^10^ | 6.19 × 10^11^ |

The detectivity (*D*) shows how much small signal can be detected by a photodetector. For accurate calculation of the detectivity, a noise spectrum is necessary to be measured. Instead, we provide the detectivity by using the simplified equation [Ref. J. M. Liu, *Photonic Devices*, Cambridge University Press, New York 2005] as follows:

$D\approx\frac{A^{\frac{1}{2}}R}{{(2qI_{dark})}^{\frac{1}{2}}}$ (2)

where *R* is the responsivity, *I*_dark_ is the dark current, and the other symbols have their usual meanings.

The calculations, summarized in Table S1, showed the detectivities of 1.02 × 10^10^ and 6.19 × 10^11^ cmHz^1/2^W^–1^ at the bias voltages of 1 V and 4 V, respectively, under 310 nm.
